# Ningnanmycin inhibits tobacco mosaic virus virulence by binding directly to its coat protein discs

**DOI:** 10.18632/oncotarget.19401

**Published:** 2017-07-19

**Authors:** Xiangyang Li, Gefei Hao, Qingmin Wang, Zhuo Chen, Yan Ding, Lu Yu, Deyu Hu, Baoan Song

**Affiliations:** ^1^ State Key Laboratory Breeding Base of Green Pesticide and Agricultural Bioengineering, Key Laboratory of Green Pesticide and Agricultural Bioengineering, Ministry of Education, Guizhou University, Guiyang 550025, P. R. China; ^2^ Key Laboratory of Pesticide and Chemical Biology of Ministry of Education, College of Chemistry, Central China Normal University, Wuhan 430079, P. R. China; ^3^ State Key Laboratory of Elemento-Organic Chemistry, Nankai University, Tianjin 300071, P. R. China

**Keywords:** ningnanmycin, TMV CP, binding analysis, interaction studies

## Abstract

Tobacco mosaic virus (TMV) causes severe plant diseases worldwide; however, effective antiviral agents for controlling TMV infections are not available. This lack of effective antiviral agents is mainly due to the poor understanding of potential targets associated with TMV infections. During infection, the coat protein (CP), which is delivered by viral particles into susceptible host cells, provides protection for viral RNA. Here, we found that Ningnanmycin (NNM), a commercially used plant antibacterial agent, inhibits the assembly of the CP by directly binding several residues. These interactions cause the disassembly of the CP from discs into monomers, leading to an almost complete loss of pathogenicity. Substitutions in the involved binding residues resulted in mutants that were significantly less sensitive to NNM. Thus, targeting the binding of viral CPs through small molecular agents offers an effective strategy to study the mechanism of NNM.

## INTRODUCTION

Plant viral diseases cause significant losses in agriculture and low-cost agents capable of effectively controlling such diseases are in great need. Antiviral compounds that target RNA and chemicals that aim at stimulating plant disease resistance have received intensive attention in recent years [1, 2]. However, few studies have focused on the use of viral coat proteins (CPs) and their assembly as potential targets for infection control [3]. Tobacco mosaic virus (TMV) causes diseases in a wide variety of economically important plants, yet few effective anti-TMV agents are available [4, 5]. Furthermore, the molecular mechanism of action for the few existing anti-TMV agents remain unclear. There is thus a great need to identify novel targets for the development of effective anti-TMV drugs [1-3,6].

The CP forms aggregate discs that interact with the newly synthesized positive sense TMV RNA to assemble progeny virions. It also facilitates the long-distance movement of the virus [7]. The aggregation of CP initiated by RNA recognition plays an important role in viral assembly, initiation and elongation. The CP is essential in maintaining and protecting the RNA genome [8]. In TMV viral particles, electrostatic interactions by residues Arg90, Arg92, Arg112 and Arg113 among monomers and with the RNA phosphate backbone are important for viral stability [9-12]. A mutational analysis revealed that residues Glu95, Glu97, Glu106, Asp109 and Asp116 [13], and the contact pair Glu50 and Asp77 in the CP were involved in the disassembly of viral particles [14]. In the absence of TMV RNA, the CP can form aggregate discs *in vitro* through physical regulation [15, 16]. The CP also affects the display of viral and host responses [17], and regulates the production of movement proteins and virus replication complexes during infection [18, 19]. In addition, CP-mediated resistance has been demonstrated using transgenic CP and hairpin RNA technology [20, 21].

The recombinant TMV CP expressed in *Escherichia coli* can assemble into discs using a hydrogen bonding network and salt bridges [6, 22, 23]. In conjunction with genomic RNA, the CP aggregates formed viral particles *in vitro* through hydrophobic and electrostatic interactions. The TMV particles artificially reconstructed from recombinant CP retain their self-assembly function [6], providing a system to identify and evaluate small molecules capable of inhibiting the CP’s functions.

Ningnanmycin (NNM) is a commonly used antimicrobial agent that exhibits an antiviral activity against TMV [24]. This compound is able to inhibit CP polymerization *in vitro* [24]. However, the mechanisms of such inhibition, as well as its anti-TMV effects, remain elusive [24]. Here, we found that NNM interferes with the assembly of the TMV CP four-layer aggregate discs. Using computational simulations and a mutational analysis, we identified the residues on the TMV CP that are directly involved in the NNM interaction. The study also establishes a useful model system that allows the rapid screening of antiviral agents against viral infections.

## RESULTS

### Identifying that NNM binds to TMV CP and TMV RNA

**Figure 1:**
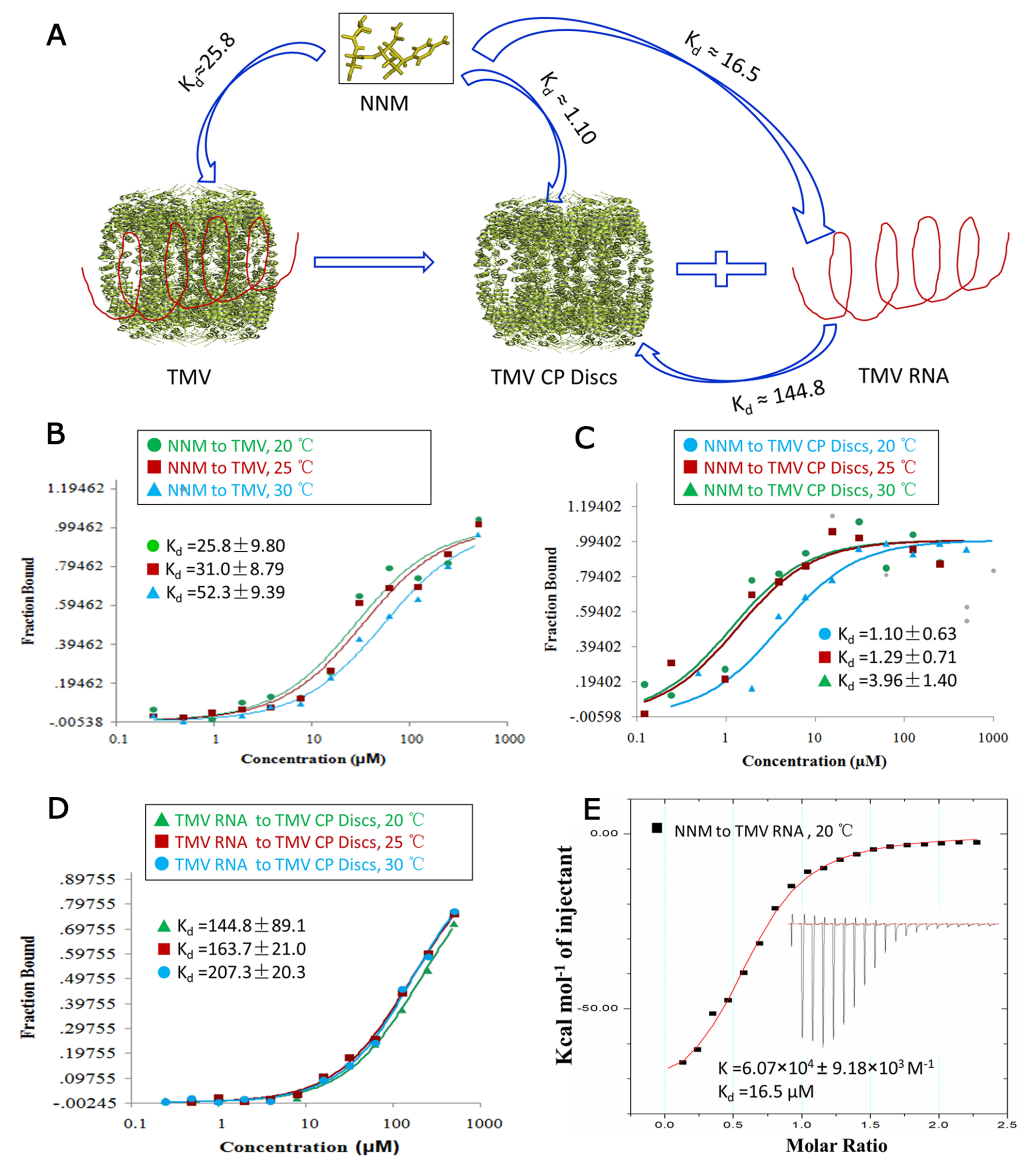
Interaction analysis of TMV–NNM, CP disc–NNM, CP disc–RNA, NNM–RNA **(A)** schematic illustration of interactions of TMV–NNM, CP–NNM, CP–RNA; **(B)** MST results showed that the Kd of NNM–TMV was 25. 8–52.3 μM from 20 to 30°C (Supplementary Table 2); **(C)** MST results showed that the Kd of NNM–CP was 1.10–3.96 μM from 20 to 30°C (Supplementary Table 2); **(D)** MST results showed that the Kd of CP –RNA was 144.8–267.3 μM from 20 to 30°C (Supplementary Table 2); **(E)** ITC results showed that six NNM combined one RNA with a Kd of 16.5 μM at 20 °C (Supplementary Table 2).

Recombinant CP four-layer aggregate discs were formed in phosphate buffer at 20°C for more than 12 h. They were collected as target proteins and TMV RNA was isolated from infected-TMV tobacco leaves. The TMV RNA was added to the recombinant CP four-layer aggregate discs to form TMV particles. The recombinant CP exhibited an activity similar to its native form in the construction of infectious viral particles [6]. We studied the interactions between NNM and TMV particles, NNM and CP discs, and TMV RNA and CP discs using microscale thermophoresis (MST) (Figure 1A). The NNM bound to TMV particles with a dissociation constant (Kd) of 25.8–52.3 μM (Figure 1B and Supplementary Table 1), the NNM bound to CP discs with a Kd of 1.10–3.96 μM (Figure 1C and Supplemental Table 1), and the TMV RNA bound to CP discs with a Kd of 144.8–207.3 μM from 20°C to 30°C (Figure 1D and Supplemental Table 1). The affinity between NNM and CP discs or TMV particles was greater than the affinity between TMV RNA and CP discs. Additionally, we studied the interactions between NNM and TMV RNA using isothermal titration calorimetry (ITC) (Figure 1E). The NNM bound to TMV RNA with a Kd of 16.5 μM (Figure 1E and Supplemental Table 1). The affinity between NNM and CP discs was greater than the affinity between NNM and TMV RNA. Thus, the target of NNM is main CP discs. Because TMV particles are composed of TMV CP discs and TMV RNA, and TMV CP discs are stabilized by numerous protein–protein salt bridges and hydrogen-bonding networks among protein layers, we reasoned that NNM bound CP with a high affinity through similar chemical bonds by competing for the residues contributing to disc stability. Thus, it may be possible to destabilize the CP complex and decrease the virulence.

### NNM induces the disassembly of TMV CP discs

**Figure 2:**
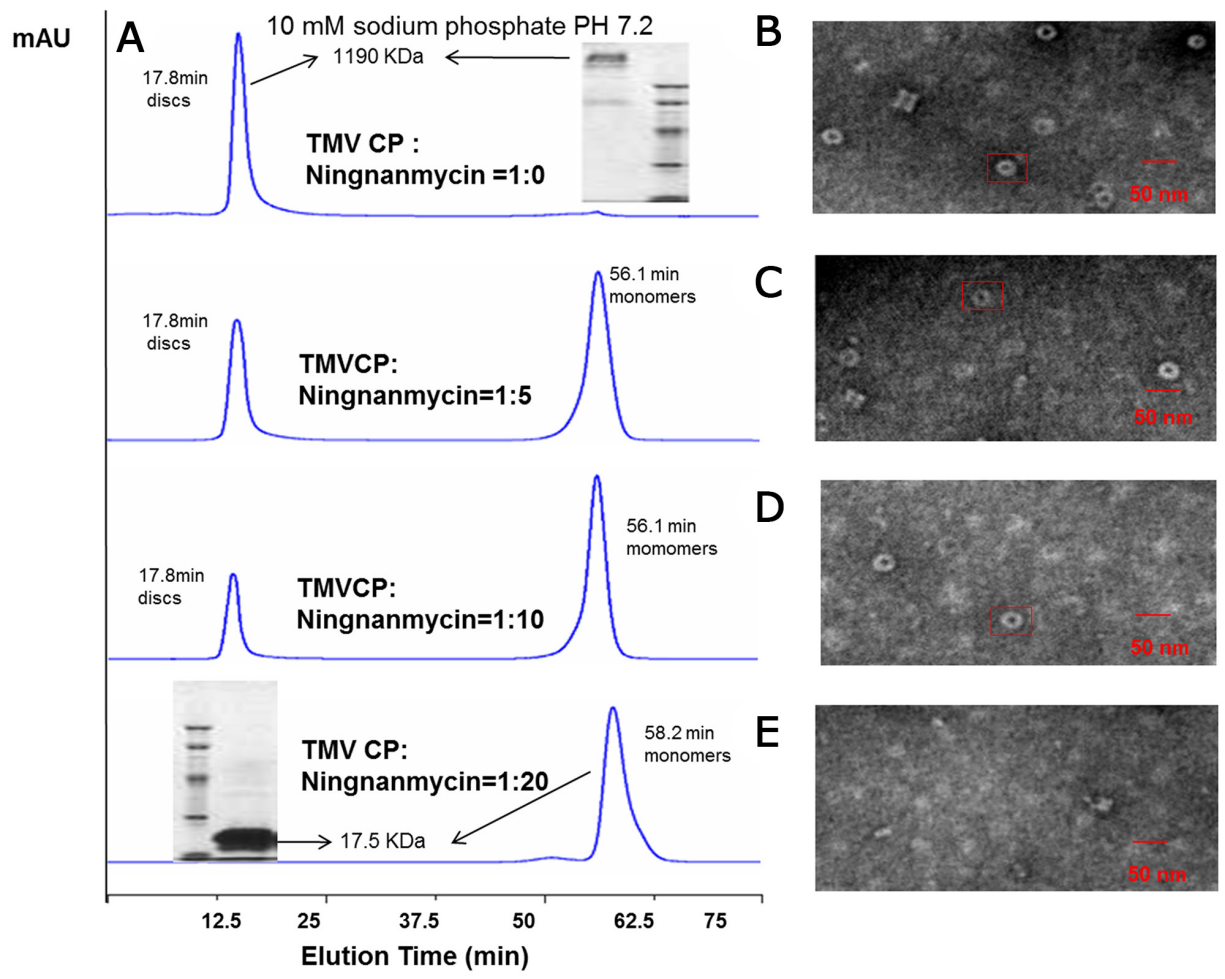
Interaction analysis of TMV CP discs–NNM. **(A)** The interactions of CP discs–NNM was analyzed using SEC. Note that the four-layer aggregate discs were eluted at 17.8 min with an approximate molecular weight of 1190 kDa, and CP monomers were eluted at 56.1–65 min with 17.5 kDa. Inserts are molecular weight estimated using native PAGE; **(B-E)** The four-layer aggregate discs in the corresponding samples visualized using TEM. Note that the four-layer aggregate discs were invisible when NNM was added to 20 times of the CP (E).

**Figure 3:**
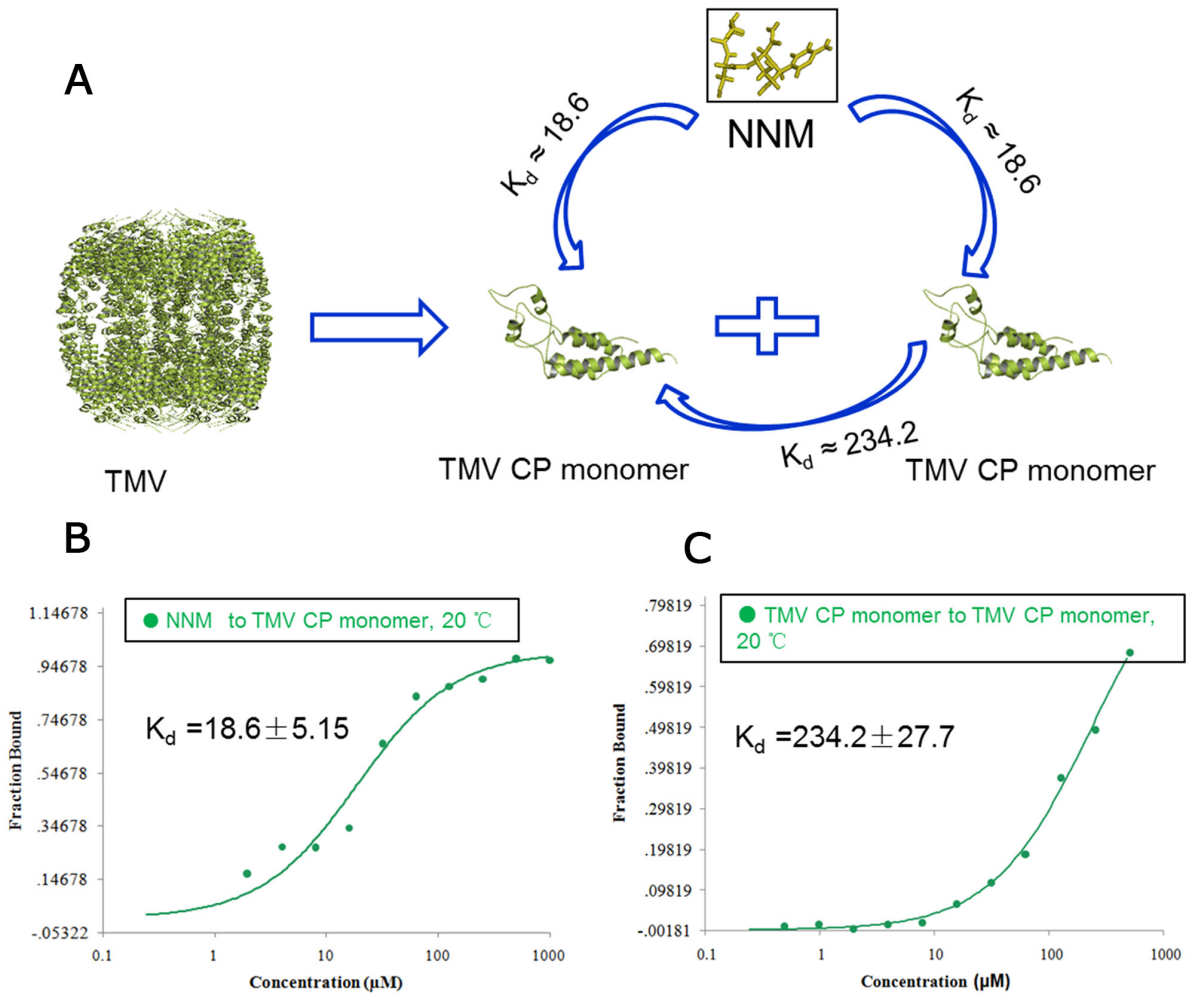
Interaction analysis of CP monomer–NNM, CP monomer–monomer **(A)**schematic illustration of the interactions of CP monomer–NNM, CP monomer–monomer; MST results showed that the Kd of NNM–CP monomer was18. 6 μM **(B)** and the Kd of CP monomer–monomer was 234.2 μM **(C)** at 20°C (Supplementary Table 2).

NNM was capable of disrupting the oligomeric structure of recombinant CPs of TMV. Briefly, recombinant TMV CP was allowed to form functional aggregates. After adding the test compounds, the protein samples were examined by size-exclusion chromatography (SEC) and transmission electron microscopy (TEM) (Figure 2). We further analyzed the effects of these compounds on the biochemical properties of this CP. As expected, initially all recombinant TMV CP was eluted as discs (Figures 2A–B). When NNM was added at a ratio of 1:5 (CP four-layer aggregate discs:NNM molecules) to the disc solution, a significant portion (60%) of the discs disassembled into monomeric proteins (Figure 2C). The induction of disc disassembly by NNM was dose-dependent. Increasing the amount of NNM led to a larger extent of disassembly. When the ratio between TMV CP and NNM reached 1:20, the discs were completely disassembled into monomeric proteins (Figures 2D–E). Additionally, the wild type (wt) CP four-layer aggregate discs (isolated from TMV particles) were used as targets, to test the interaction between the wt CP four-layer aggregate discs and NNM. When the ratio between the wt CP four-layer aggregate discs and NNM molecules reached 1:20, the wt CP four-layer aggregate discs were completely disassembled into monomeric proteins (Supplementary Figure 1). The affinities between NNM–CP monomers and CP–CP monomers were evaluated, and the results implied that NNM have a strong affinity of 18.6 μM, leading to CP disc disassembly (the Kd of CP–CP was 234.2 μM) (Figures 3A–3C). We thus deduced that NNM disrupts the uniform multimeric structure of the CP.

**Figure 4:**
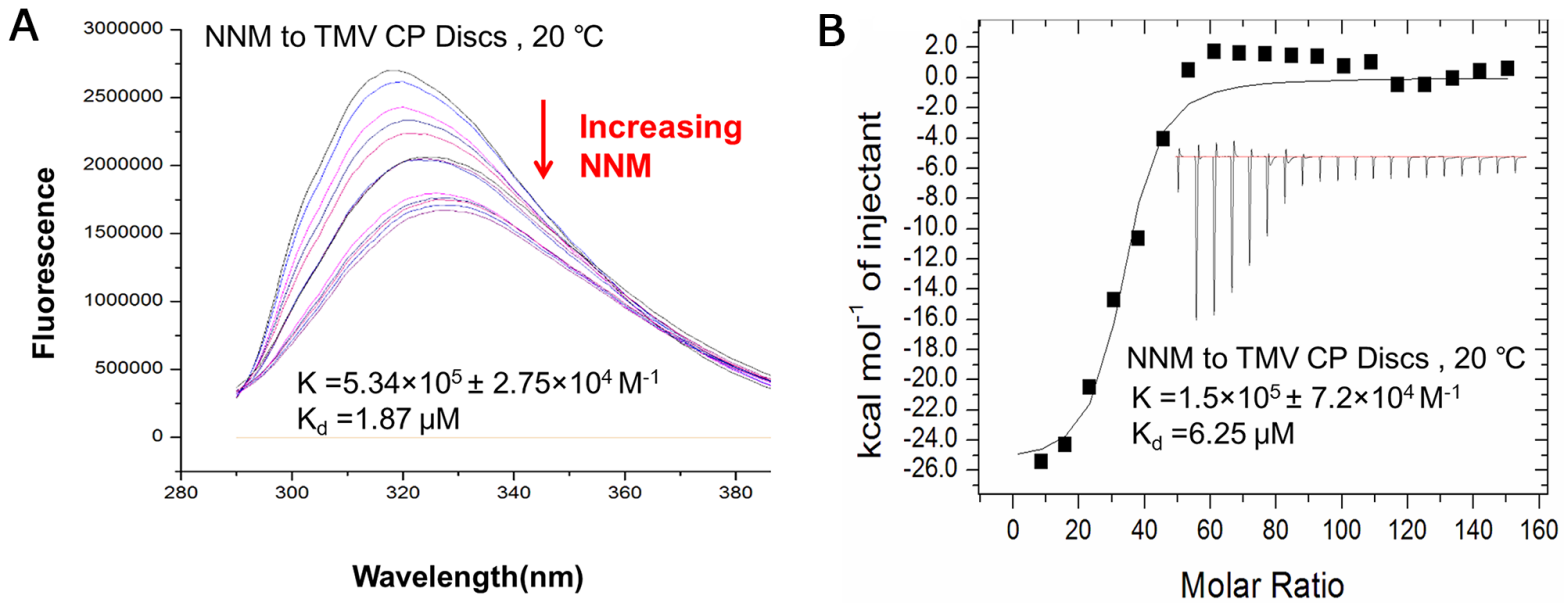
Interaction analysis of NNM–CP disc using fluorescence and ITC **(A)** Fluorescence measurements showed that the Kd of NNM–CP disc was 1.87 μM (Supplementary Table 2); **(B)** ITC results showed that one CP disc combined 39–41 NNM with a Kd of 6.25 μM (Supplementary Table 2).

We next determined the affinity between CP and NNM using two other independent methods, fluorescence spectrum and ITC. Fluorescence spectrum measurements showed that the NNM bound to TMV CP discs with a Kd of 1.87 μM (Figure 4A and Supplementary Table 2). The analysis by ITC revealed that one CP disc interacted with 39 to 41 NNM molecules, and NNM bound to CP discs with a Kd of 6.25 μM (Figure 4B and Supplementary Table 2). The titration data (ΔH=17200 cal/mol and ΔS=−79.1 cal/mol/deg) also indicated an apparent negative enthalpy value (ΔG = −6.0) during the binding of NNM to the TMV CP monomers (Figure 4B), indicating that the CP−NNM complex is stable.

### Molecular dynamics (MD) simulation

**Figure 5:**
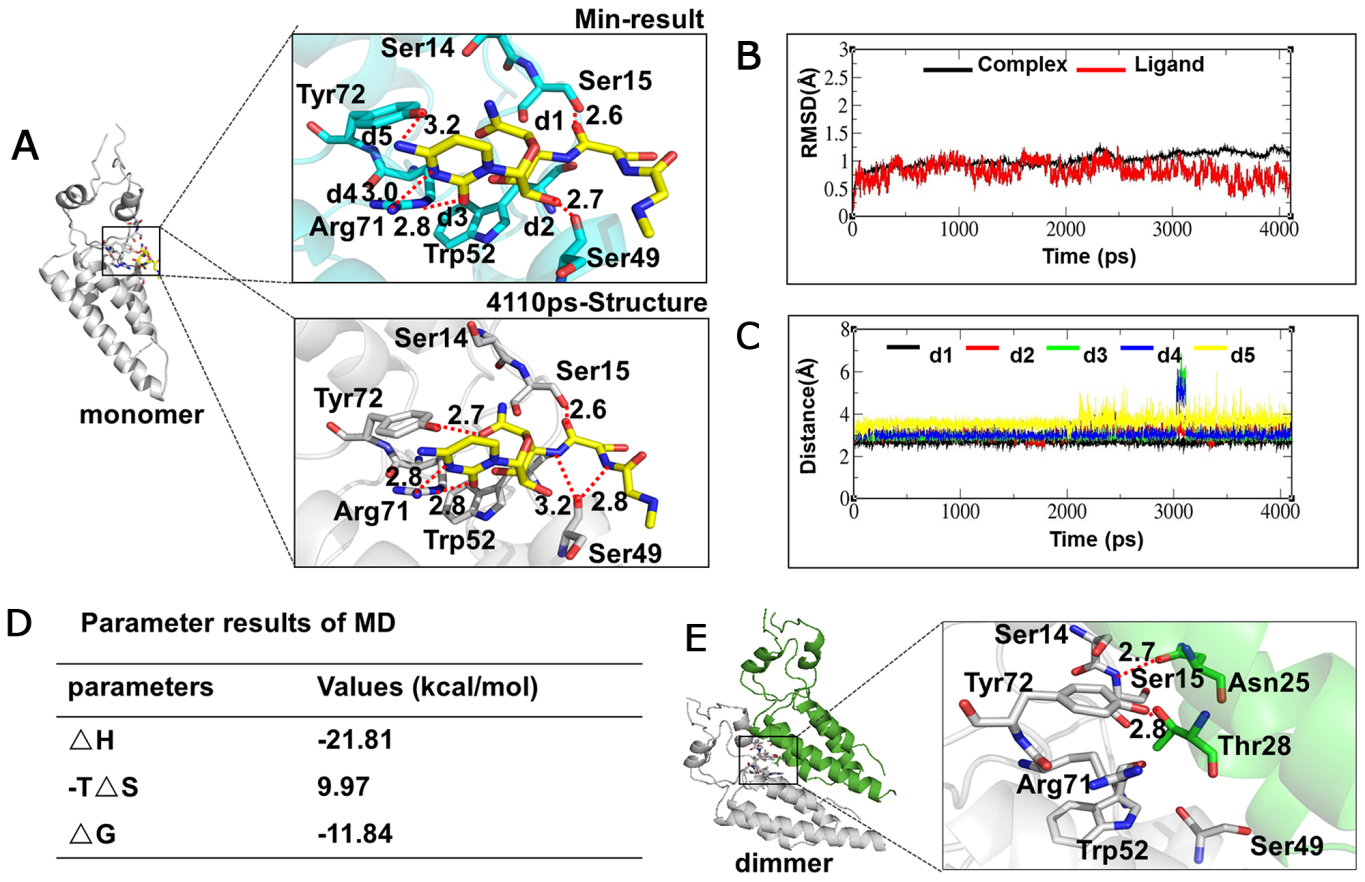
Interaction analysis of NNM–CP monomer and CP monomer–monomer using MD **(A)** Conformational analysis of CP monomer–NNM; **(B)** Dynamics trajectory analysis of NNM–monomer; **(C)** Critical distance analysis of NNM–monomer; **(D)** Dynamic trajectory data of NNM–monomer at the last 1ns; **(E)** Conformational analysis of CP monomer–monomer.

To identify the NNM recognition sites in the TMV CP, we performed molecular docking using the Gold method with 200 cycles. Six binding sites involving Ser14, Ser15, Ser49, Trp52, Arg71 and Tyr72 were found in the representative conformations (Supplementary Figure 2, conformations 1 to 6). After optimization, conformations 3 and 6 were considered as the optimum (Supplementary Figures 2C and 2F). In conformation 3, five strong hydrogen bonds involving Ser15 (one H bond with NNM), Ser49 (one), Arg71 (two) and Tyr72 (one) can be formed between the CP and NNM (Figure 5A and Supplementary Figures 3A–C). However, in conformation 6, three strong hydrogen bonds involving Ser15 (one), Ser49 (one) and Arg71 (one) were formed (Supplementary Figures 3D–F). Trajectory dynamics and a critical distance analysis revealed that both conformations 3 (Figures 5B–C and Supplementary Figures 4A–E) and 6 (Supplementary Figures 4E–H) are stable during MD simulations. There are more hydrogen bonds formed between the small molecule and the CP in conformation 3, with four stronger hydrogen bond interactions observed in the final state’s structure. However, fewer hydrogen bonds were formed between the small molecule and CP in conformation 6, with three hydrogen bond interactions observed in the final state’s structure. As a result, in conformation 3, the electrostatic interaction between the CP and NNM was great, indicating that was optimum conformation potentially formed by NNM and the TMV CP monomer (Figures 5A–C). Using conformation 3 as an example, five hydrogen bonds involving Ser15, Ser49, Arg71 and Tyr72 were formed between the CP and NNM at the beginning of the MD simulations. The strengths and numbers of hydrogen bonds involving Ser15, Ser49, Arg71 and Tyr72 did not significantly change during the MD simulations (Figure 5D).

We also performed MD simulations of the interactions between two adjacent CP monomers. Only two hydrogen bonds, Ser15–Asn25 and Tyr2–Thr28 were found by molecular docking (Figure 5E). Additionally, the MD simulation analysis showed that the interactions among the CP monomers were stable in the initial structure with approximately 1 Å fluctuations at the final state (4,110 ps) of structural dynamics (Supplementary Figure 5). Taken together, these analyses revealed that the binding of NNM to the CP has a greater affinity than those seen in the interactions among CP monomers in the formation of dimers, oligomers or discs. Such a difference underlies the capability of NNM to induce the disassembly of CP discs.

### Mutational analysis of the protein–ligand interaction between NNM and CP

**Figure 6:**
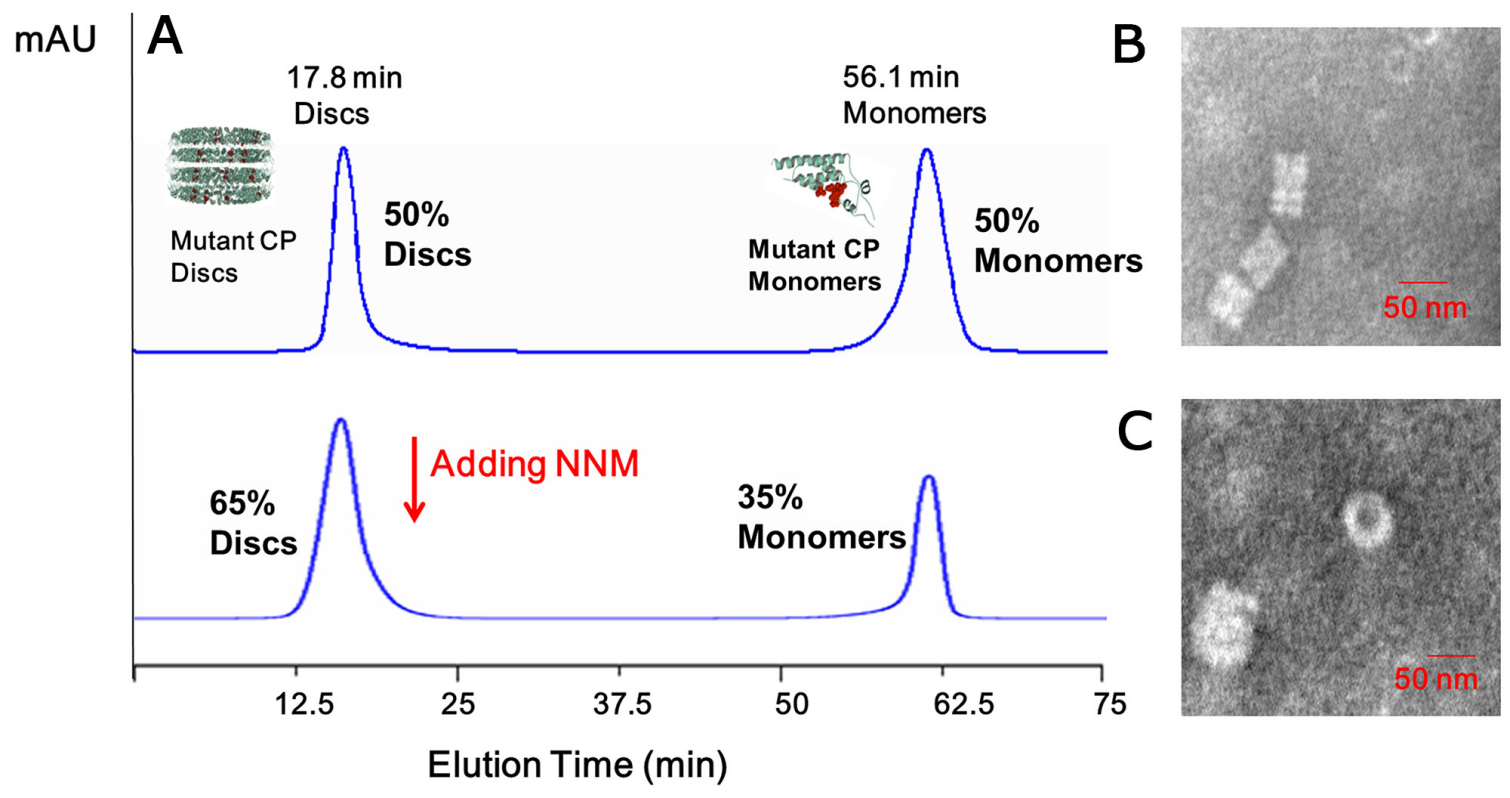
Analysis of the interactions of mutated CP–CP and mutated CP disc–NNM **(A)** About 50% mutated CP discs and 50% mutated CP monomers were visible using SEC; when adding the excessive NNM, about 65% mutated CP discs and 35% mutated CP monomers were visible in 10 mM sodium phosphate buffer using SEC. The presence of the discs was evaluated using TEM, mutated CPs **(B)** and mutated CP–NNM **(C)**.

**Figure 7:**
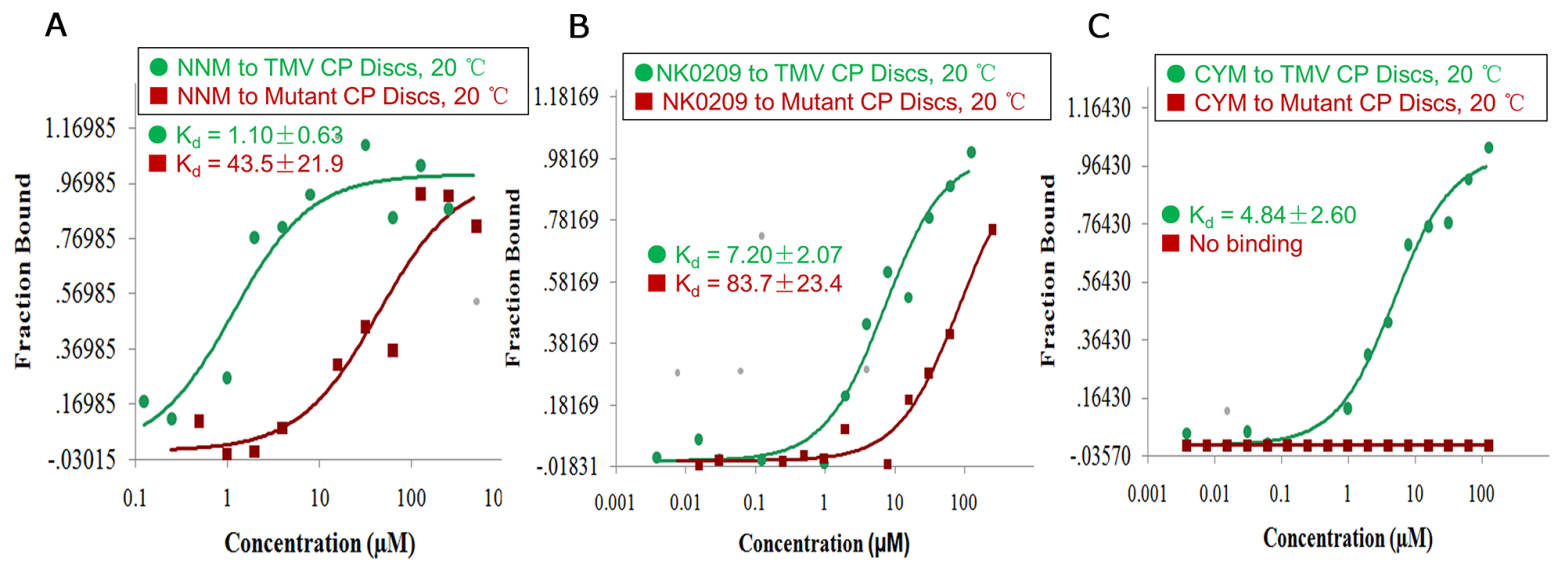
Binding analysis of NNM–CP, NK0209–CP and cytosinpeptidemycin–CP **(A)** MST results showed that the Kd of NNM–CP monomer was 1.10 μM, the Kd of NNM–mutated CP disc was 43.5 μM at 20°C (Supplementary Table 2); **(B)** MST results showed that the Kd of NK0209–CP monomer was 7.20 μM, the Kd of NK0209–mutated CP disc was 83.7 μM at 20°C (Supplementary Table 2); **(C)** MST results showed that the Kd of cytosinpeptidemycin–CP monomer was 4.84 μM, there is no binding between cytosinpeptidemycin and the mutated CP disc, which is in good agreement with the binding results obtained using fluorescence and ITC (Supplementary Table 2).

To analyze the roles of the residues in the CP identified in the simulations as being critical for binding NNM, we constructed and expressed four CP mutants, S15G, S49G, R71G and Y72G. SEC and TEM revealed that approximately 50% of the mutated CPs assembled into discs (Figures 6A–B). When NNM was added at a 1:20 (CP discs:NNM) ratio, only approximately 35% of mutated protein discs disassembled into protein monomers (Figures 6B–C). Furthermore, an analysis by MST revealed that the Kd of an NNM–mutant CP disc was approximate 43.5 μM (Figure 7A), which was weaker than the NNM–CP disc (Figure 1C). The Kd of NNM–mutant CP monomer was approximate 211.8 μM (Supplemental Figure 6A), which was weaker than the NNM–CP monomer (Figure 3B). The Kd of mutant CP monomer–monomer was approximate 989.6 μM (Supplementary Figure 6B), which was weaker than the CP monomer–monomer (Figure 3C).Thus, the four residues interacted with NNM in a competitive manner.

### Evaluating NNMs ability to inhibit TMV replication *in vivo*

**Figure 8:**
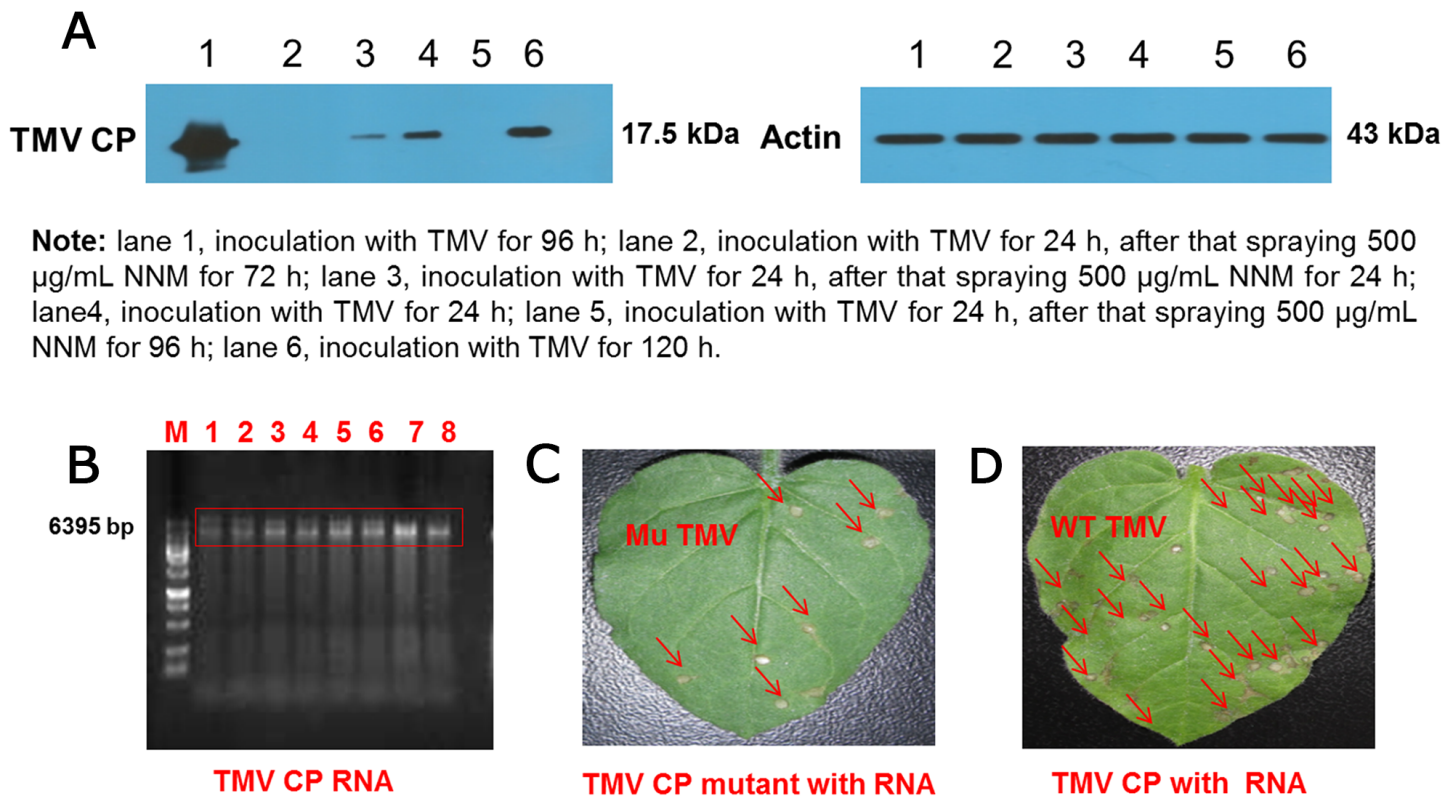
Western blot analysis of inhibition activities of NNM against TMV CP replication and verification of the virulence of TMV reconstituted particles from wt CP and mutated CP **(A)** The bands of CP were weak when treated with 500 μg/mL NNM, the molecular weight of CP is 17.5 kDa, and the actin control was added with 43 kDa; **(B)** TMV RNA integrity (lanes 1–8) was examined by 1% agarose gel electrophoresis; **(C)** Leaves of *N. glutinosa* were inoculated with the reconstituted TMV derived from mutated CP. Local lesions caused by mutated TMV were labeled with red arrows, note the less severe symptoms; **(D)** The *N. glutinosa* was inoculated with the reconstituted wt TMV, the local lesions induced by the reconstituted wt TMV were labeled with red arrows.

To assess whether NNM can inhibit TMV CP replication in a systemic infection of the host *Nicotiana tabacum cv. K*_*326*_ *(N. tabacum cv. K*_*326*_*),* the leaf disc method along with western blot analysis, of TMV CP exposed to 0.5 mg/mL NNM for 24–96 h were carried out. The bands of the CP were weak when sprayed with 0.5 mg/mL NNM for 24 h and the bands of the CP were not visible after 72 h (Figure 8A), which indicated that the newly assembled TMV virions in tobacco were inhibited by NNM Additionally, we collected the mutated protein discs and reconstituted a protein solution of 1.7 mg/mL, which was incubated with 0.5 mg/mL TMV RNA for 30 min to reconstitute viral particles (Figure 8B). In TMV inoculation assays, the infectivity of the viruses derived from mutated CP significantly decreased (Figure 8C). Additionally, the mean lesions caused by the mutant viruses were about one quarter the size of those caused by the full-length reconstituted virus (Figure 8D). Thus, residues Ser15, Ser49, Arg71 and Tyr72, which were identified in computational simulations, indeed play key roles in the NNM–CP interactions.

### The use of CP discs as targets to screen anti-TMV compounds

The effects of NNM suggest that the CP of TMV is a potential anti-viral drug target. Thus, the effects of several compounds on the properties of the CP were tested. In total, 0.5 mM (8.7 mg/mL) of fresh TMV CP discs were mixed independently with 5 mM NK0209 or cytosinpeptidemycin. Reactions with NNM were used as controls in all of the assays. The Kd values of treatments with compounds NK0209 or cytosinpeptidemycin bound to TMV CP discs and their mutants are listed in Supplementary Table 2. The affinities of the compounds to the mutant CP were weaker than the affinities of the compounds to the full-length CP, indicating that the mutation sites in the CP are the key residues (Figures 7B–7C). Based on compounds being capable of binding CP directly, CP can be regarded as anti-TMV compound target. Thus, we screened anti-TMV compounds and found that compounds NK0209 and cytosinpeptidemycin were capable of binding CP directly. Furthermore, NK0209 and cytosinpeptidemycin exhibited significant anti-TMV activities in 500 μg/mL and had very good controlled effects (Supplementary Table 3) against TMV-associated disease in a vivo experiment. Our results support a model in which the disruption of the CP structure begins with the binding of NNM to CP within the four-layer CP aggregate. This binding leads to the disassembly of the CP discs into monomers in complex with the small molecules. Traditionally, the prevention of infection, by measures such as the use of virus-free seedlings, has been considered the only effective way to control plant viral diseases. The ability of NNM to induce the disassembly of CP discs suggests that this compound may effectively cure established TMV infections.

## DISCUSSION

To explore the CP of TMV as a potential drug target, we took advantage of recombinant CP being similarly active to its native form by examining the effects of small molecules on the disassembly of CP discs. Our efforts led to the identification of NNM as a potent agent that induced the disassembly of TMV CP discs. To the best of our knowledge, this is the first example demonstrating that the targeting of a viral CP’s physiochemical properties by small molecular agents is an effective strategy in controlling diseases caused by plant viruses.

The interactions between NNM and TMV CP monomers were mediated by at least five strong hydrogen bonds, which were significantly stronger than those that mediated the interactions among CP monomers. The interactions among individual subunits in protein complexes are normally dynamic, and the complexes can be seen as one end of the equilibrium. By binding to CP discs with a greater affinity, NNM could tilt the balance toward complexes formed between the CP and the molecule itself, leading to the disruption in the disc’s structure.

Our results support a model in which the disruption of the CP structure begins with the binding of NNM to CP in the four-layer CP aggregate. Such binding leads to the disassembly of the CP discs into monomers complexed with the small molecules. Traditionally, the prevention of infection, by measures such as the use of virus-free seedlings, was considered the only effective means for controlling plant viral diseases [27]. The ability of NNM to induce the disassembly of CP discs suggests that this compound may effectively cure established TMV infections.

The notion that NNM functions by competing with residues involved in hydrogen bonds, which are responsible for the interactions among CP monomers, was further supported by the observation that mutations in residues (S15G, S49G, R71G, and Y72G) may impair the sensitivity of CP to NNM-induced disassembly. For example, residues Ser15 and Tyr72 are critical for the formation of hydrogen bonds among CP monomers. Yet, both residues are involved in the binding to NNM. Clearly, the competition for binding sites contributes to the effects of NNM. Further, the fact that viral particles containing the mutant CP are less virulent is in line with their roles in stabilizing the CP. Finally, our results also provide evidence indicating that CP plays an important role in the pathogenesis of TMV.

The interactions of the test compounds NK0209 and cytosinpeptidemycin with TMV CP mutated discs reduced the binding ability of CP mutants as assessed by MST, which was in good agreement with the binding results obtained by fluorescence spectrum and ITC. The value of using CP as a target for anti-viral agent development was further validated by the identification of several additional compounds that are capable of binding CP directly and reducing TMV virulence. In summary, our results demonstrate that TMV CP discs could be used as a new model target to screen for anti-TMV compounds. Because CP assembly is a critical step in the maturation of many viruses, including those of animals, our method could, in principle, be used to identify agents that act against viruses that pose threats to human health or agricultural production.

## MATERIALS AND METHODS

### Materials

NNM was kindly provided by Chen Jia-ren, Chengdu Institute of Biology, Chinese Academy of Sciences. NK0209 was synthesized by Wang Qing-min of the Research Institute of Elemento-Organic Chemistry. Cytosinpeptidemycin was bought from Fujian Quanzhou biopharmaceutical companies (Supplementary Table 4).

### Constructing recombinant TMV CP

To prepare TMV CP discs, TMV was purified using the method described by Gooding and modified by Shire [25, 26]. TMV RNA was extracted from the purified virus by treating with phenol and sodium dodecyl sulfate (SDS) [27, 28]. TMV RNA was reverse transcribed using the previously described primers in 50 mmol/L Tris solution at pH 8.0, containing 8.0 mmol/L magnesium chloride, 75 mmol/L potassium chloride, 10 mmol/L DL-dithiothreitol, 1.0 mmol/L dNTPs, 0.5 unit/μL AMV reverse transcriptase (TaKaRa, Shiga, Japan), and 1.0 unit/μL RNase inhibitor (TaKaRa) for 1.5 h at 42 °C to generate the full-length viral cDNA sequence [6]. Following the generation of the full-length viral cDNA, the coding region of the TMV CP was amplified by PCR [6]. dsDNA of the full-length was purified and analyzed by 1% agarose gel electrophoresis. pET28a (Novagen, Madison, WI, USA) and TMV CP were digested using Nde I (NEB, Hertfordshire, England, 10 units/μL)/Xho I (NEB, 10 units/μL) and cloned into similarly digested pET28a. *Escherichia coli* strain BL21 (DE3)-plysS (Novagen) was transformed with the aforementioned recombinant plasmid. Cultures for protein expression were grown in Luria-Bertani medium containing 30 μg/mL kanamycin at 37°C until the optical density at 600 nm reached 0.7. After cooling the cultures to 16°C, the expression was induced by adding isopropyl β-D-1-thiogalactopyranoside to 0.5 mmol/L, and induction was allowed to proceed for 16 h. The cells were harvested by centrifugation and resuspended in 40 mL lysis buffer (100 mmol/L sodium chloride and 50 mmol/L phosphate buffer [pH 8.0] and 10 mmol/L *β*-mercaptoethanol). The cells were thawed, lysed using a supersonic device, and centrifuged at 13,400 ×g for 30 min at 4°C. After passing through a 0.22-mm syringe filter (Millipore, Schwalbach, Germany), the supernatant was loaded onto a Ni^2+^ Sepharose High-Performance Column (GE Healthcare, Piscataway, NJ, USA), washed with five column volumes of 40 mmol/L imidazole and eluted with 400 mmol/L imidazole. The elute was concentrated using an Amicon Ultra centrifugal filter device (Millipore) with a 10 kDa MW filter and then loaded onto a HiLoad 16/60 Superdex 200 pg column equilibrated with a dialysis solution (10 mM sodium phosphate buffer and 100 mM sodium chloride solution [pH 7.2]). After thrombin digestion of 6×His tags at 4°C overnight, TMV CP was further purified by SEC using a Superdex 200 10/300 GL column (GE Healthcare) and a buffer containing 10 mM sodium phosphate and 100 mM sodium chloride solution (pH 7.2). Proteins were then concentrated to 8.7 mg/mL for biochemistry experiments using amicon ultra centrifugal filter units (Millipore) with a 10 kDa MW cutoff. The target proteins were briefly stored at 4°C. TMV (common strain) was isolated from infected tobacco leaves (grown in the greenhouse at Guizhou University) and purified [25, 26]. The CP was separated and purified using the modified acetic acid degradation method and dialyzed against the appropriate high-salt buffer at room temperature to obtain the four-layer aggregate [6].

### Functional determination of recombinant TMV CP

The recombinant proteins with 6×His tags were purified using Ni^2+^ beads and the tags were cleaved using thrombin. TMV CP was examined for the formation of oligomers with SEC methods. Discs were formed by incubating the oligomers in 10 mM sodium phosphate buffer containing 100 mM sodium chloride (pH 7.2) at 22°C for more than 12 h [6]. TMV CP discs were detected by native PAGE after TMV CP oligomers were incubated at 22°C for 24 h, and the formation of four-layer aggregate discs was confirmed by SEC. The refolding and further self-assembly of TMV CP were performed with a protein concentration of 8.7 mg/mL incubated at 22°C for 24 h, and the four-layer aggregate discs were observed by TEM. To reconstitute TMV particles, the same concentration of CP was incubated with 2 mg/mL TMV RNA at 22°C for 24 h. The freshly purified TMV CP oligomers that self-assembled into TMV CP discs could be reconstructed into newly infectious viruses [6].

### Interactions between NNM and TMV CP

NNM and TMV CP binding was initiated by adding 5 mM anti-TMV compounds to 0.5 mM (8.7 mg/mL) TMV CP discs and incubating for 1 h. Then, 0–10 mM NNM was added to the reactions for 1 h. The oligomer status of TMV was then analyzed by SEC [6]. SEC was performed at room temperature using a calibrated Superdex 200 10/300 GL column (GE Healthcare) attached to an AKTA Purifier Fast Protein Liquid Chromatography system (GE Healthcare). The column was equilibrated with a buffer containing 10 mM sodium phosphate and 100 mM sodium chloride solution (pH 7.2). The molecular mass standards (Bio-Rad Hercules, CA, USA) used included thyroglobulin (669 kDa), ferritin (440 kDa), Bovine Serum Albumin (67 kDa), *β*-lactoglobulin (35 kDa), ribonuclease A (13.7 kDa), cytochrome (13.6 kDa), aprotinin (6.51 kDa) and vitamin B12 (1.36 kDa). The protein was monitored by measuring the absorbance at a wavelength of 280 nm. For TEM [6, 22, 23], self-assembled TMV CP discs were incubated as described previously. Briefly, 20 μL of the mixed solution was deposited onto a 300-mesh formvar-carbon-coated copper grid for 2 min, followed by rinsing with ddH_2_O. The grid was stained with 20 μL of 2% aqueous solution of tungstophosphoric acid for 90 s as a negative stain. Images were obtained at the Electron Microscope Lab of Zunyi Medical University using a Hitachi H-7650 transmission electron microscope (Tokyo, Japan) with 80 kV accelerating voltage. The 17% native PAGE [29-31] was performed on ice with TMV CP samples equilibrated overnight in a buffer containing 10 mM sodium phosphate and 100 mM sodium chloride (pH 7.2). Then, 20 μL of the samples was mixed with 20 μL of 2× loading buffer (12.5% 0.5 M Tris-HCl (v/v) (pH= 8.7), 0.5% bromophenol blue (w/v) and 30% glycerin (v/v). Subsequently, 8 μL of the samples was loaded onto 17% gels. Electrophoresis was performed using a 1× native PAGE buffer (Tris-Gly, pH 8.8) at 0 °C for 1 h. After electrophoresis, gels were stained with Coomassie Brilliant blue to identify proteins, and then destained with methanol and glacial acetic acid. The fluorescence spectra [32] were recorded with a fluorescence spectrophotometer (Varian Cary Eclipse, Palo Alto, CA, USA) at 20°C. The emission spectra of TMV CP discs were obtained in buffer (1.5 mL) with a quartz cuvette having a 1-cm path length. Fluorescence intensities were measured with an excitation wavelength of 278 nm and an emission wavelength of 325 nm. The concentrations of TMV CP discs were defined at 50 nM. NNM was continuously added into the cuvette until the fluorescence signal no longer changed. All measurements were taken in phosphate buffer (10 mM sodium phosphate, 100 mM sodium chloride [pH 7.2]), and fluorescence titration curves were corrected for the background intensity of the buffer. The apparent dissociation constants were analyzed by the nonlinear least-squares curve-fitting method using Origin 7.0 software. The ITC binding experiments [33] were performed using an ITC 200 Micro Calorimeter (GE Healthcare) at 20°C. All recombinant CP proteins and mutants were formed in 10 mM sodium phosphate buffer and 100 mM sodium chloride solution (pH 7.2) at 20°C for more than 12 h, and then recombinant CP four-layer aggregate discs and mutant discs were collected. The buffer contained 10 mM sodium phosphate and 100 mM sodium chloride (pH 7.2). The compounds (0–10 mM) were titrated into TMV CP discs and mutants (0.5 mM) in a 200 μL sample cell using a 40-μL microsyringe as follows: 0.4 μL for the first injection and 2 μL for the next 19 injections at intervals of 150 s. The integrated heat data were analyzed using the one-set-of-sites model in MicroCal Origin 7.0 according to the manufacturer’s instructions. The first data point was not used in analysis. The binding parameters reaction enthalpy change in cal·mol^−1^ (ΔH), binding constant in mol^−1^ (K) and the number of molecules per TMV CP proteins (n) were floating during the fit. The binding free energy, ΔG, and reaction entropy, ΔS, were calculated using the equations, ΔG = −RTlnK (R = 1.9872 cal·mol^−1^·K^−1^, T = 298 K) and ΔG = ΔH−TΔS. The Kd was calculated as 1/K. Then, the binding was calculated for MST [34, 35] Monolith NT. 115 (Nano Temper Technologies, Munchen, Germany). A range of ligands from 0 μM to 5 μM were incubated with 0.5 μM of purified recombinant proteins for 5 min with a NT-647 dye (Nano Temper Technologies). These were used in the thermophoresis experiment at a final concentration of ∼20 nM. A 16 point dilution series was made for selected compounds in dimethyl sulfoxide. Each compound’s dilution series was subsequently transferred to protein solutions in 10 mM Tris/HCl and 100 mM sodium chloride, pH 7.4, containing 0.05% Tween-20. After a 15 min incubation of the labeled CP with each dilution point (1:1 mix) at room temperature, samples were placed into standard capillaries (NanoTemper Technologies). Measurements were taken on a Monolith NT.115 MST (NanoTemper Technologies) under the setting of 20% LED and 40% IR laser. The laser’s on time was set at 30 s, and the laser’s off time was set at 5 s. The Kd values were calculated from the duplicate reads of three separate experiments using the mass action equation in the Nano Temper software. For western blot [36], an electrotransfer system (Bio-Rad) was used. Growing leaves of *N. tabacum cv.* K_326_ were mechanically inoculated with equal volumes of TMV (0.5 mg/mL). After 72 h, 1-cm diameter leaf discs were removed. The leaf discs were floated on solutions of NNM and on buffer (10 mM sodium phosphate and 100 mM sodium chloride solution, pH 7.2) as a negative control. Discs of healthy leaves were floated on buffer as a mock. All of the leaf discs were kept in a culture chamber at 28°C for 48 h, and then, the TMV concentration in the leaf disc was determined. Leaf discs were ground in 5 × protein loading buffer (10% SDS, 5% *β*-ME, 50% glycerin, 0.5% bromophenol blue, and 250 mM Tris-HCl, pH 6.8), and then, 5 μL of sample were loaded on a polyacrylamide gel (5% stacking gel and 12% separating gel). After SDS-PAGE, TMV protein bands were transferred at 90 mA for 1 h onto a polyvinylidene fluoride membrane (0.46 μm, washed with methanol to activate) using an electrotransfer system (Bio-Rad). The membrane was washed in TBST (20 mM Tris-HCl, pH 8.0; 150 mM NaCl; and 0.05% Tween-20) and blocked with 5% nonfat milk powder in TBST for 1 h at 37°C. The membrane was washed three times, each time for 3 min with TBST, and reacted with a mixture of 1:30,000 alkaline phosphadase-conjugated anti-rabbit IgG (Sigma, Deisenhofen, Germany) and 1:200 polyclonal antibody of TMV for 2 h at 37°C. After it was washed three times, each time for 3 min with TBST, the membrane was incubated in substrate buffer (12.1 g Tris-HCl, pH 9.5; 5.84 g NaCl; 10.2 g MgCl_2_; and 800 mL H_2_O) with 330 μL/mL nitrotetrazolium blue chloride and 165 μL/mL 5-bromo-4-chloro-3-indolyl phosphate for 3–5 min in the dark until the bands of the CP were clear. For the MD simulation, the X-ray crystal structure of TMV CP was downloaded from (Protein Data Bank) PDB (PDB ID: 4GQH). The initial structure was revised by adding lost residues and hydrogen atoms and checking bonds and bumps. Subsequently, the energy was minimized for 2,000 steps of the steepest descent calculations and 2,000 steps of conjugated gradient calculations by using Sybyl 7.0 (Tripos Inc., St. Louis, MO, USA) and Gaussian03 program at the HF/6-31+G* level [37]. The optimized geometries were used to construct the entire structures. The final structures of different conformations were optimized by fixing the macrocycle with a conjugated gradient in Sybyl 7.0. The different conformations were used as the starting structures for docking studies. Docking calculations were performed on these conformations with AutoDock4.0. The protein and ligand structures were prepared with Autodock Tools [38]. The atomic Gasteiger–Huckel charges were assigned to the ligand and receptor. Most of the parameters for the docking calculation were set to the default values recommended by the software. Each docked structure was scored by the built-in scoring function and was clustered by 0.8 Å of (root-mean-square deviation) RMSD criteria. For each binding model, the molecular mechanics/Poisson–Boltzmann surface area was calculated. Before this calculation, the complex structure was further refined initially with the steepest descent algorithm, followed by the conjugated gradient algorithm using the Amber9 package [39]. During the energy minimization process, the receptor was first fixed, and only the ligand remained free. Then, the ligand and residue side chains remained free. Finally, all atoms of the system were liberated and refined to a convergence of 0.01 kcal/(mol·Å).

Based on the docking results, two binding models were selected for MD simulation. Prior to the simulation, the electrostatic potential and partial atomic charges were determined by performing an electrostatic potential fitting according to the Merz–Singh–Kollman scheme with the Gaussian-optimized geometries [40, 41]. The rescaled electrostatic potential charges of the ligand were produced using the standard protocol implemented in the antechamber module of the Amber9 program [38, 42, 43]. The system was solvated in an octahedral box of TIP3P water, in which crystallographic water molecules were maintained. The edge of the box was at least 10 Å from the solute. Appropriate sodium counterions were added to the system to preserve neutrality. The solvated system belonged to the solute. Before the MD simulation, some energy minimization steps were applied to the system. First, the solute was kept fixed with a constraint of 500 kcal mol^−1^Å^−2^. Water and counterions were minimized. The backbone atoms of the protein were then fixed with the ligand, side chains, and other atoms that are free to move. Finally, the entire system was fully minimized without any constraint. In each step, energy minimization was first performed using the steepest descent algorithm for 2,000 steps, and subsequently, the conjugated gradient algorithm was used for another 3,000 steps. The MD simulation was performed under periodic boundary conditions using the sander module of the Amber9 program. First, the system was fixed to heat only the water and counterions for 10 ps to make sure the solute was fully solvated. Then, the entire system was gradually heated from 10 K to 300 K using the weak-coupling method and equilibrated for 100 ps with the protein backbone fixed [44]. Lastly, the system was switched to a constant pressure equilibration (2 ns). During the MD simulation, the particle mesh Ewald algorithm was used to handle long-range electrostatic interactions with a cutoff distance of 10 Å [45, 46], which was also used for the van der Waals energy terms. All of the angles and bonds involving hydrogen atoms were constrained using the SHAKE algorithm [47]. The time step used for the MD simulations was 2.0 fs, and the coordinates were collected every 1 ps.

### Verifying the interaction between NNM and mutated TMV CP

To verify the results of the MD simulation, four mutations [14], S15G, S49G, R71G, and Y72G, were introduced into the CP, and the mutant proteins were used to measure the interactions between NNM and TMV CP as described previously (see interactions between NNM and TMV CP). Then, 1mL of purified self-assembled mutant discs (8.7 mg/mL) were incubated in 10 mM sodium phosphate and 100 mM sodium chloride solution, pH 7.2, and mixed with 0.2 mL purified TMV RNA (2 mg/mL). The mixture was incubated at 20°C for 24 h. Suspensions were centrifuged at 2,700 ×g for 1 min, and reconstituted viruses were obtained [6].

Leaves of *Nicotiana glutinosa (N. glutinosa)* were mechanically inoculated with the reconstructed full-length virus or the reconstructed mutated virus (see the TEM methods). The local lesion numbers were recorded 3–4 days after inoculation.

## SUPPLEMENTARY MATERIALS TABLES



## Abbreviations

TMV: tobacco mosaic virus
CP: coat protein
NNM: ningnanmycin
MST: microscale thermophoresis
Kd: dissociation constant
ITC: isothermal titration calorimetry
SEC: size-exclusion chromatography
TEM: transmission electron microscopy
Wt: wild type
*N. tabacum cv. K*_*326*_: *Nicotiana tabacum cv. K*_*326*_
PDB: protein data bank
RMSD: root-mean-square deviation
RESP: rescaled electrostatic potential
*N. glutinosa*: *Nicotiana glutinosa*
