# Supplementary material for: Ningnanmycin inhibits tobacco mosaic virus virulence by binding directly to its coat protein discs
Source: Oncotarget. 2017 Jul 19;8(47):82446–58. doi: 10.18632/oncotarget.19401 (PMC5669903; doi:10.18632/oncotarget.19401)
Supplement: Supplementary file 1 [file oncotarget-08-82446-s001.pdf]

Ningnanmycin inhibits tobacco mosaic virus virulence by binding directly to its coat protein discs

SUPPLEMENTARY MATERIALS

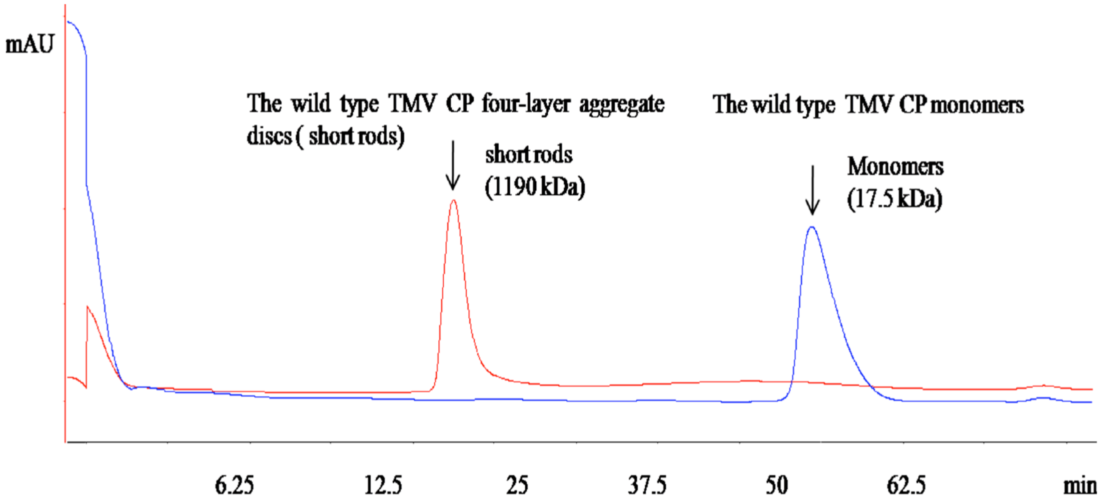

Supplementary Figure 1: Interaction analysis of wt TMV CP disc–NNM. 1 mM wt TMV CP four-layer aggregate discs incubated with 20 mM NNM was analyzed using SEC by blue solid line, the wt TMV CP discs was analyzed using SEC by red solid line. Note that fresh wt CP discs was collected as target proteins to study the interactions of wt CP disc–NNM. The wt CP discs were eluted at 17.8 min with an approximate molecular weight of 1190 kDa, and wt TMV CP monomers, which is disassembled by NNM, were eluted at 56.1 min with an approximately molecular weight of 17.5 kDa.

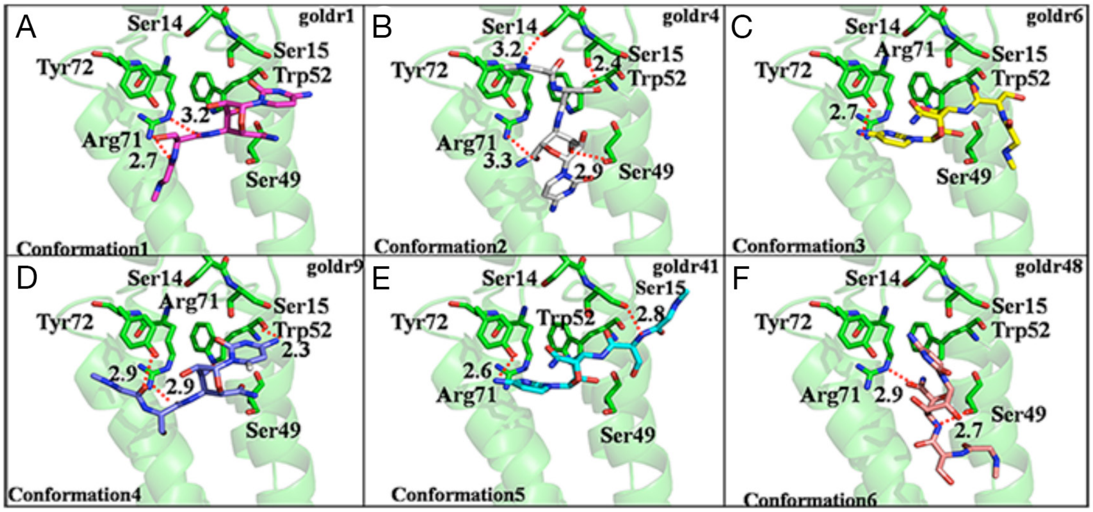

Supplementary Figure 2: Conformational analysis of TMV CP monomers was performed to obtain a total of 6 conformations, which was used as the starting structure for docking with NNM. From the six representative conformations, hydrogen bonds are present between Arg71 and NNM in conformation 1 **(A)**, between Ser14, Ser15, Ser49, Arg71 and NNM in conformation 2 **(B)**, between Arg71, Tyr72 and NNM in conformation 3 **(C)**, between Trp52, Arg71, Tyr72 and NNM in conformation 4 **(D)**, between Ser15, Arg71, Tyr72 and NNM in conformation 5 **(E)**, and between Ser49, Arg71and NNM in conformation 6 **(F)**.

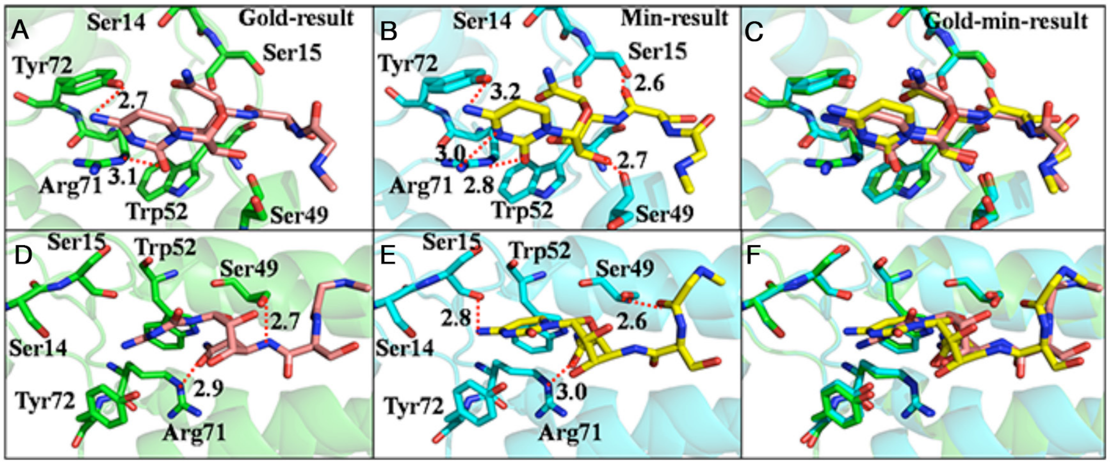

Supplementary Figure 3: Optimization conformations 3 and 6 between NNM and TMV CP monomer. Prior to the optimization, there were only two hydrogen bonds between Arg71, Tyr72 and NNM in conformation 3 **(A)**, after the optimization, five strong hydrogen bonds were detected between Ser15, Ser49, Arg71, Tyr72 and NNM **(B)**, the superposition of crystal structure diagram between conformation 3 and its optimization were shown in **(C)**. The same as conformation 6, before the optimization, there were only two hydrogen bonds between Arg71, Ser49 and NNM in conformation 6 **(D)**, after the optimization, three strong hydrogen bonds were formed between Ser15, Ser49, Arg71 and NNM **(E)**, the superposition of crystal structure diagram between conformation 6 and its optimization were shown in **(F)**.

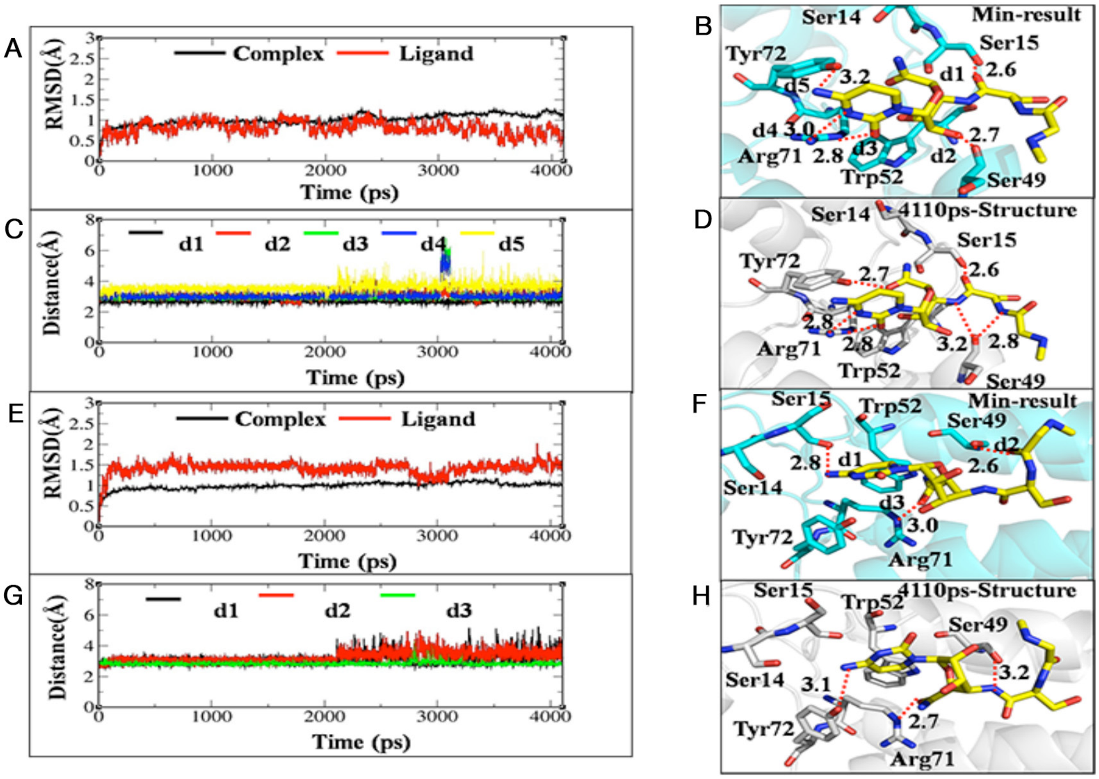

Supplementary Figure 4: Dynamics trajectory and critical distance analysis of the conformations 3 and 6 between NNM and TMV CP monomer. Compared to the initial structure, the TMV CP monomer in conformation 3 was stable in the entire process of the MD simulations, with about 1 Å fluctuations; and the small molecule NNM was also stable, with about 0.5–1 Å fluctuations **(A)**; for the complex structure of conformation 3, at the beginning of the MD simulations, five hydrogen bonds **(B)** were formed between Ser15, Ser49, Arg71, Tyr72 and NNM, to set the distance limit in the first 2 ns, and do open restrictions after 2 ns, in the whole process, five key distance were stable **(C)**; In the process of the MD simulations, there was a little structure change between the small molecules and the CP which caused the loss of some hydrogen bonds and the formation of some new one at the end of the MD simulations (4110 ps), four hydrogen bonds were detected between Ser15, Ser49, Arg71, Tyr72 and NNM in conformation 3 **(D)**. In the same way, the dynamic process of conformation 6 was monitored by RMSD trajectories and the critical distance, and the conformational change of the dynamic process was analyzed. Compared with the initial structure, TMV CP monomer in conformation 6 was stable in the process of the entire MD simulations, with about 0.9 Å fluctuations; and small molecule NNM was also stable, with about 1.5 Å fluctuations **(E)**, for the complex structure of conformation 6, at the beginning of the MD simulations, three hydrogen bonds between Ser15, Ser49, Arg71 and NNM were detected **(F)**; to set the distance limit in the first 2 ns, and do open restrictions after 2 ns, in the whole process, three key distance were stable **(G)**; the complex structure of conformation 6 was also stable. At the end of the MD simulations (4110 ps), three new hydrogen bonds between Ser49, Arg71 were detected between Tyr72 and NNM in conformation 6 **(H)**.

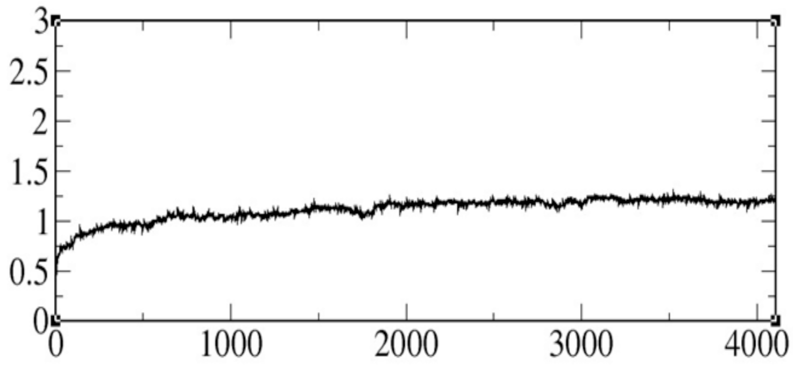

Supplementary Figure 5: Dynamics trajectory of the TMV CP monomer–monomer. Compared to the initial structure, the TMV CP monomer–monomer was stable in the process of the entire MD simulations, with about 1 Å fluctuations.

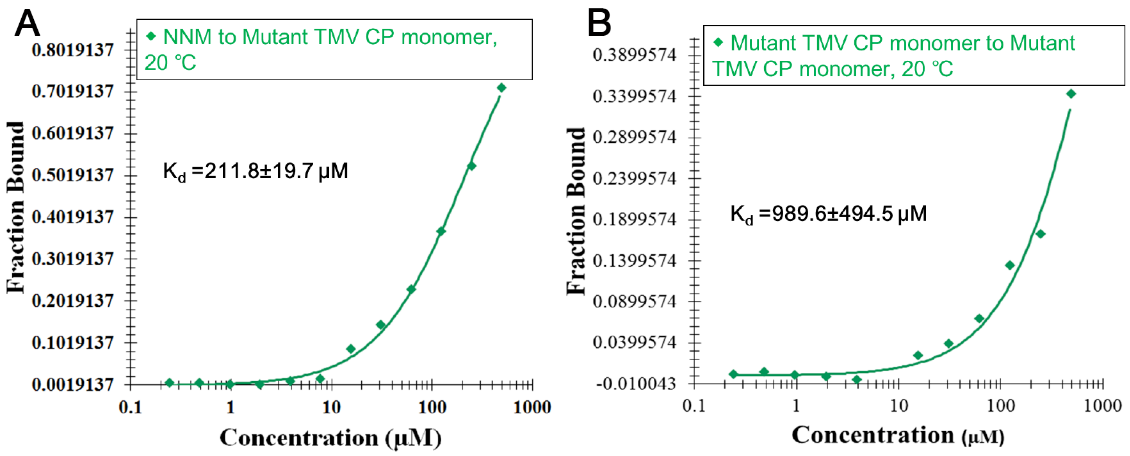

Supplementary Figure 6: MST results showed that the Kd of the mutant TMV CP monomer–NNM was 211.8 µM **(A)**, and the Kd of the mutant TMV CP monomer–monomer was 989.6 µM **(B)** at 20°C.

Supplementary Table 1: TMV particles, RNA and CP Proteins binding studied with NNM using MST and ITC[a]

Ligands	TMV particles (MST, µM)	CP discs (MST, µM)	CP monomers (MST, µM)	TMV RNA (ITC, µM)
NNM	25.8 (20°C); 31.0 (25°C); 52.3 (30°C)	1.10 (20°C); 1.29 (25°C); 3.96 (30°C)	18.6 (20°C)	16.5 (20°C)
RNA	no datas	144.8 (20°C); 163.7(25°C); 207.3(30°C)	no datas	no datas
CP monomer	no datas	no datas	234.2 (20°C)	no datas

[a]The MST data were fitted with NTAffinity Analysis software; the ITC data were fitted to a one-set-of-sites model.

Supplementary Table 2: TMV CP Proteins and its mutant binding studied using fluorescence spectrum, ITC and MST

Compounds[b]	$Kd_{Proteins}$ (Fluo., µM)	Kd_{mutant} (Fluo., µM)	$Kd_{Proteins}$ (ITC, µM)[c]	Kd_{mutant} (ITC, µM)[c]	$Kd_{Proteins}$ (MST, µM)	Kd_{mutant} (MST, µM)
NNM	1.87	1428	6.25	220	1.10	43.5
NK0209	1.25	25.6	31.2	133	7.20	83.7
Cytosinpeptidemycin	23.2	250	5.41	33.0	4.84	No binding

[b]The experiment was performed with 5 mM compounds and 0.5 mM TMV CP.

[c]The ITC data were fitted to a one-set-of-sites model.

Supplementary Table 3: The results of curative activity against TMV of the antivirial compounds

Compounds	Curative activity against TMV (100 µg/mL, %)[d]	Curative activity against TMV (500 µg/mL, %)[d]
Ningnanmycin	29.8	60.0
NK0209	30.8	71.5
Cytosinpeptidemycin	11.9	48.7

[d]The experiment was inoculated by means of half leaf.

Supplementary Table 4: The structural formula of the antivirial compounds[e]

Drugs name	Molecular formula
Ningnanmycin	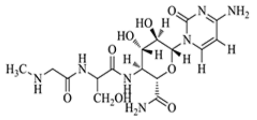
NK0209	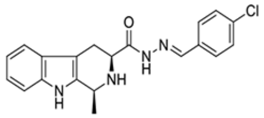
Cytosinpeptidemycin	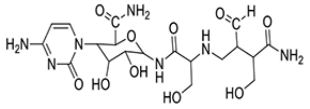

[e]Pure product.
